# Highlights of the 14th International *Bordetella* Symposium

**DOI:** 10.1128/msphere.00189-25

**Published:** 2025-05-16

**Authors:** Kevin Munoz Navarrete, Kathryn M. Edwards, Kingston H. G. Mills, Jana Kamanová, María Eugenia Rodriguez, Andrew Gorringe, Andrew Preston, Beate Kampmann, Monica C. Gestal, Eric T. Harvill, Purnima Dubey, Dimitri A. Diavatopoulos, Seema Mattoo, Karen M. Scanlon, Camille Locht, Peter Sebo

**Affiliations:** 1 Institute of Microbiology of the Czech Academy of Sciences, Prague, Czechia; 2 Division of Pediatric Infectious Diseases, Department of Pediatrics, Vanderbilt University Medical Center 12328, Nashville, Tennessee, USA; 3 School of Biochemistry and Immunology, Trinity Biomedical Sciences Institute, Trinity College Dublin 8809, Dublin, Leinster, Ireland; 4 CINDEFI (UNLP, CONICET La Plata), Facultad de Ciencias Exactas, Universidad Nacional de La Plata 28228, La Plata, Buenos Aires Province, Argentina; 5 UK Health Security Agency, Porton Down, Salisbury, United Kingdom; 6 The Milner Centre for Evolution and Department of Life Sciences, University of Bath 1555, Bath, United Kingdom; 7 Clinical Research, London School of Hygiene & Tropical Medicine 364969, London, United Kingdom; 8 Institute of International Health, Charité - Universitätsmedizin Berlin 425077 , Berlin, Germany; 9 Department of Microbiology and Immunology, Louisiana State University Health Sciences Center at Shreveport 547965, Shreveport, Louisiana, USA; 10 Department of Infectious Diseases, University of Georgia College of Veterinary Medicine 70734, Athens, Georgia, USA; 11 Department of Microbial Infection and Immunity, The Ohio State University 683676 , Columbus, Ohio, USA; 12 Laboratory of Medical Immunology, Radboud Community of Infectious Diseases, Radboud Institute for Molecular Life Sciences, Radboud University Medical Center 691252, Nijmegen, Gelderland, Netherlands; 13 Departments of Biological Sciences and Biochemistry, Purdue University 311308 , West Lafayette, Indiana, USA; 14 Department of Microbiology and Immunology, University of Maryland School of Medicine, Baltimore, Maryland, USA; 15 Université Lille, Centre National de la Recherche Scientifique, Inserm, Centre Hospitalier Universitaire Lille, Institut Pasteur de Lille, U1019 Unité Mixte de Recherche 8204, Center for Infection and Immunity of Lille, Lille, France; University of Kentucky College of Medicine, Lexington, Kentucky, USA

**Keywords:** *Bordetella pertussis*, vaccines, epidemiology, pathogenesis, virulence, toxins

## Abstract

Pertussis, or whooping cough, is a highly contagious and acute respiratory illness caused primarily by the gram-negative coccobacillus *Bordetella pertussis*. Despite near-universal vaccination, pertussis remains one of the least-controlled vaccine-preventable infectious diseases. Since 2023, pertussis incidence has been rising, and widespread pertussis outbreaks have resurged in many countries. In response to these emerging challenges, almost 300 experts from institutions across 24 countries convened at the 14th International *Bordetella* Symposium in Prague, Czech Republic, from 24 to 28 June 2024 to discuss pertussis epidemiology and research and strategies to mitigate the global pertussis burden. We present here the highlights of the symposium, comprising epidemiological and clinical aspects of *Bordetella* infections, results of clinical trials of pertussis vaccination in pregnant women and effectiveness of maternal vaccination in protecting newborn infants in Africa and Europe, the controlled human infection model (CHIM), and the latest insights into the biology, immunology, and pathogenesis of *B. pertussis* infection.

## INTRODUCTION

The genus *Bordetella* encompasses 16 species of gram-negative coccobacilli that are found in the environment or as pathogens associated with a variety of respiratory diseases of animals and humans. Of these, three so-called “classical *Bordetella*” species—*B*. *pertussis*, *Bordetella parapertussis,* and *Bordetella bronchiseptica*—are of public and animal health relevance (1). While human-adapted *B. parapertussis*_HU_ can also cause whooping cough, pertussis toxin (PT)-producing *B. pertussis* is the primary causative agent of pertussis. The severe respiratory symptoms of pertussis were the leading cause of infant mortality before the global introduction of pertussis vaccines. Despite the availability of life-saving vaccines, pertussis still remains a global health burden, estimated to account for about 160,000 deaths annually among children under 5 years of age and for about 20 million cases of whooping cough, which ranks pertussis among the least controlled vaccine-preventable pediatric infectious diseases (2). Finally, *B. bronchiseptica*, a zoonotic pathogen, occasionally infects animal breeders or immune-compromised humans and is responsible for a wide range of diseases, including bronchopneumonia and atrophic rhinitis in piglets, snuffles in rabbits, kennel cough in dogs, and bronchitis in cats (3).

*B. pertussis*, *B. parapertussis*, and *B. bronchiseptica* produce an overlapping repertoire of virulence factors, whose production is mainly regulated at the transcriptional level by the *bvgAS* locus. This two-component system consists of the sensor kinase BvgS and the response regulator BvgA (4, 5). Among these *Bordetella* virulence factors, several facilitate adhesion to host airway epithelial cells, including filamentous hemagglutinin (FHA) and fimbriae types 2 and 3 (FIM2/FIM3) (1, 6). Following adhesion, pertactin (PRN), the *Bordetella* colonization factor A (BcfA), and tracheal cytotoxin (TCT) contribute to respiratory tract colonization (7–9). Other virulence factors modulate or facilitate evasion of host immune responses: BrkA, BapC, Vag8, and exopolysaccharide inhibit complement activation on *B. pertussis* (10); adenylate cyclase toxin (ACT) subverts both innate and adaptive immunity through its cell-invasive adenyl cyclase enzyme activity (11); and PT, produced exclusively by *B. pertussis,* hijacks cellular G protein-coupled receptor signaling, dysregulates immune responses, and mediates systemic effects, such as leukocytosis, hyperinsulinemia, and histamine sensitivity (12–14). To establish infection, the classical *Bordetella* species also utilize the Type III secretion system (T3SS), a molecular apparatus mediating direct delivery of effector proteins into the host cell cytosol, with the cytotoxic effector protein BteA/BopC being the most significant *Bordetella* effector (15–17).

Currently, there are two general types of pertussis vaccines available: the whole-cell pertussis vaccine (wPV), containing entire heat- or formaldehyde-killed *B. pertussis* bacteria and presenting a wide range of bacterial antigens and TLR ligands; and the acellular pertussis vaccine (aPV), consisting of up to five purified *B. pertussis* protein antigens (18). The wPV was approved for use in combination with diphtheria and tetanus toxoids (DTwP) in the 1940s (19) and, once incorporated into the infant immunization programs, it led to a dramatic >99% reduction in pertussis cases. For instance, in the United States, the number of reported cases dropped from approximately 270,000 before the introduction of the wP vaccine to just 1,010 cases by 1976 (20).

Despite its high efficacy, wPVs were associated with occasional significant systemic reactions, which prompted the development of the less reactogenic aPVs (21). The aPVs all contain chemically or genetically modified pertussis toxoid, with or without other antigens such as FHA, PRN, and FIM2/3 (22). Over time, various formulations of the aPV have been developed, with differing antigen compositions, adjuvants, purification techniques, and detoxification methods (23).

The recent resurgence of whooping cough outbreaks in most aP-vaccinated populations of high-income countries remains a topic of debate. Many believe the primary factor has been the transition from the wPV to aPV. wP vaccines contain a diverse array of antigens and TLR ligands that induce Th1-/Th17-polarized immunity, while the less reactogenic aPV confer predominantly Th2-polarized immunity and provide a shorter duration of immunity (24). While aPVs are effective in preventing severe pertussis disease, they are less efficient in preventing *B. pertussis* colonization, allowing the pathogen to circulate more easily and cause higher rates of symptomatic infection within the population (25–28).

### PERISCOPE (PERtussis Correlates of Protection Europe) Project vaccination studies

During the first day of the International *Bordetella* Symposium, key findings of the PERISCOPE (Pertussis Correlates of Protection Europe) project (https://periscope-project.eu/) were presented. The project coordinator, Dimitri Diavatopoulos (Radboud University Medical Centre, Nijmegen, The Netherlands), introduced the PERISCOPE project, a public-private partnership, spanning from 2016 to 2022 and seeking to accelerate next-generation pertussis vaccine development (29). A central objective of PERISCOPE was the determination of immunological biomarkers of protection against pertussis in human vaccination studies and in human and animal challenge models for *B. pertussis* infection. An overview of the various clinical studies and of the biomarker discovery platform was provided.

As part of the PERISCOPE consortium, clinical trials in pregnant women and their infants were conducted in Finland, the United Kingom, and The Gambia (30–32). The aim was to better understand the impact of maternal vaccination on primary infant immune responses to Expanded Program on Immunization (EPI) vaccines and to generate data on the reactogenicity and immunogenicity of wPV and aPV in infants. Qiushui He (University of Turku, Turku, Finland) presented the results of an open-label study conducted by Finnish researchers. They enrolled 47 mother-infant pairs where the mothers had been vaccinated during pregnancy, along with 27 pairs where mothers had not been vaccinated during pregnancy. All infants in the study were subsequently immunized with aPV. Dominic Kelly (University of Oxford, Oxfordshire, UK), Beate Kampmann and Anja Saso (MRC Unit The Gambia at the London School of Hygiene & Tropical Medicine, London, UK), and Janeri Fröberg (Radboud Centre for Infectious Diseases, Nijmegen, The Netherlands) reported the results of two mother/infant studies, with one conducted in the United Kingdom, where pertussis vaccination during pregnancy is a national recommendation, and the second conducted in The Gambia, West Africa, where pertussis vaccination is not part of the national program for pregnant women. In the United Kingdom, researchers enrolled 114 infants born to mothers who had all received aPV during pregnancy. These infants were randomized to receive either primary immunization with either aPV or wPV. At the MRC Unit in The Gambia, 343 women were enrolled in a double-blind, randomized controlled trial to receive either the aPV or the tetanus toxoid (TT) vaccine during pregnancy. Their newborns were also randomized to receive either aPV or wPV, as part of the routine EPI vaccine schedule. Per-protocol results from 239 mother-infant pairs were presented, including data on mucosal immune responses. The three studies consistently demonstrated the following key findings.

- Vaccination during pregnancy was well tolerated, with minimal reactogenicity and excellent immunogenicity observed among pregnant women.
- Transplacental transfer of antibodies was highly efficient.
- Blunting of anti-PT antibody responses was observed in infants born to mothers aP-vaccinated during pregnancy. This effect was more pronounced in infants who received wPV compared to those who received aPV.
- Quantitative antibody responses varied across the antigens included in pertussis vaccines, while qualitative responses, measured using serum bactericidal and antibody deposition assays, were less affected. The impact on qualitative responses was notably minimal when infants received the wPV in the United Kingdom or the aPV in The Gambia. However, differences in vaccine types and schedules between the countries likely contributed to observed variations.
- Maternally derived pertussis-specific IgG was detected in the infant nasal mucosa. While blunting was also shown at this level, it could be mitigated by subsequent wP vaccination in infants after the third vaccine dose.
- The clinical significance of these quantitative and qualitative immunogenicity differences, particularly at the mucosal surface, remains to be determined in terms of their effectiveness in preventing infection and disease.

This session also highlighted novel strategies for measuring immune responses alongside studies that evaluated specific immune components in *B. pertussis* human challenge studies as well as in studies with convalescent and vaccinated individuals. Jacques Van Dongen (Leiden University Medical Center, Leiden, The Netherlands) described the strategies of the EuroFlow consortium (https://euroflow.org/) for dissecting the differentiation, maturation, and activation pathways of the B-cell and plasma cell compartment following vaccination or *B. pertussis* human challenge. Expansion and maturation of IgG1+ plasma cells were observed 7 days post-vaccination (33), and early plasma cell expansion was observed in *B. pertussis-*challenged individuals who did not develop detectable colonization (34). Cecile van Els (National Institute for Public Health and the Environment, Bilthoven, The Netherlands) described approaches to characterize antigen-specific T-cell responses following vaccination. A novel semi-high-throughput whole blood assay was developed to detect *B. pertussis-*specific Th1, Th2, and Th17 responses (35, 36), which showed reduced CD4^+^ T-cell responses in older age. A simplified whole blood assay was also developed to quantify T-cell cytokines in response to *B. pertussis* antigens, demonstrating that memory T-cell responses were influenced by the primary vaccine background (37).

Andrew Gorringe (UK Health Security Agency, Porton Down, Salisbury, UK) presented a comparison between antibody responses across PERISCOPE clinical studies. In infants, wPV induced greater toxin neutralization activity per unit of anti-PT-IgG compared to aP vaccines. Sera from pertussis-convalescent individuals contained higher anti-PT IgG titers compared with most vaccinated individuals. In addition, sera from convalescent individuals or those immunized with wPV also had higher serum bactericidal activity (SBA), mediated by multiple antigens, than that induced with aPV pertactin alone, generating SBA following aPV vaccination. Janeri Fröberg showed that infection, rather than aP vaccination, elicited high concentrations of serum IgA capable of binding whole *B. pertussis*. IgA levels increased with age in sera from Dutch children, suggesting widespread *B. pertussis* exposure within these age groups.

### Human volunteer infection studies

Among the key objectives of the PERISCOPE Project was the development of a human volunteer challenge model of *B. pertussis* infection. Robert Read (University of Southampton, Southampton, UK) introduced the PERISCOPE controlled human infection model (CHIM) studies (Phase A–C) conducted at the University Hospital Southampton (38). He briefly discussed the standardization of the optimal dose for the pertussis human model, which involved an intranasal inoculation of 10⁵ colony-forming units (CFU) of the European *B. pertussis* isolate BP1917 to achieve a colonization rate of 80%. He also emphasized the ethical considerations involved in working with human challenge models. Diane Gbesemete (University of Southampton, Southampton, UK) summarized the main findings from phase A, which determined the inoculum dose that induces at least 70% colonization in a serologically selected participant population. Subsequently, phase B encompassed a follow-up outpatient study to investigate the safety of the model in a serologically unselected population. Fifty participants were challenged and included in the per-protocol analysis, with 26 undergoing a re-challenge 3 months later. All participants were treated with azithromycin at day 14 after challenge. The colonization rate after the primary challenge was 40%, and primary exposure significantly reduced the colonization rate and bacterial density following the secondary challenge. Adverse events were mostly mild, with no significant differences between colonized and non-colonized participants. Additionally, transmission was evaluated in fourteen contact volunteers, i.e., bedroom sharers, but no transmission of *B. pertussis* was detected, possibly because the challenged subjects were vaccinated as infants with wPV and were repeatedly infected asymptomatically throughout their life, having built-up mucosal immunity to pertussis.

Hans de Graaf (University of Southampton, Southampton, UK & Radboud University Medical Centre, Nijmegen, The Netherlands) presented antibody data from Phase B of the human volunteer challenge study. Antibody analysis revealed that non-colonized participants had higher pre-inoculation levels of serum IgG and IgA against *B. pertussis* vaccine antigens, as well as higher nasal IgA against whole *B. pertussis* bacteria, compared to participants who became colonized upon infectious challenge. Among the colonized volunteers, baseline serum and nasal IgG levels to *B. pertussis* vaccine antigens were inversely associated with total bacterial load, while no such association was observed with IgA. Post-challenge, significant increases in IgG and IgA were observed only in colonized participants, suggesting that colonization has an immunizing effect. Alison Hill (University of Southampton, Southampton, UK) presented cellular immunity data from Phase B, including memory B cell (MBC) and T cell responses. No differences were observed in the baseline frequency of IgG-producing MBCs against *B. pertussis* between colonized and non-colonized participants. However, in colonized participants, the frequency of baseline IgG-producing MBC was inversely associated with bacterial density. Consistent with the antibody data, higher IgG-producing MBCs were only observed in colonized participants post-*B. pertussis* challenge. Finally, a potential role for Th22 responses in protection against colonization was identified. Diane Gbesemete then introduced Phase C, an extended colonization study investigating natural clearance for up to 6 weeks post-challenge.

May ElSherif (Dalhousie University, Halifax, Nova Scotia, Canada) provided an in-depth description of the establishment of the North American controlled human infection model (*B. pertussis* CHIM) in Canada. The dose escalation and confirmation study phases involved 66 volunteers, of whom the younger 39% were aP-vaccinated and the older 61% were wP-vaccinated. The study analyzed controlled *B. pertussis* infection of volunteers with challenge doses increasing up to 5 × 10^7^ CFU of the American clinical *B. pertussis* isolate D420. From day 6 post-challenge, bacterial shedding, as defined by the detection of bacteria in nasal samples (nasopharyngeal aspirates or nasal washes) by PCR or culture, was analyzed, and correlates of infection were determined. A correlation between the challenge dose and degree of bacterial shedding was observed, and, following challenge, bacterial shedding was found to be significantly higher in aP-vaccinated participants compared to the wP-vaccinated participants. Moreover, about 72.7% (16 out of 22) of participants who received the target dose of 10^7^ CFU developed mild catarrhal pertussis disease symptoms, with fatigue and malaise starting by day 7 post-challenge, nasal congestion and runny nose on days 7 to 9, sore throat by day 10, with a higher proportion of the symptomatic participants in the aP-vaccinated cohort compared to the wP-vaccinated cohort. Cough developed from days 10 to 12 in approximately one-third of participants who had four or more catarrhal pertussis stage disease symptoms. However, no fever or severe respiratory symptoms were reported, and azithromycin treatment effectively reduced infection within 48 hours. Levels of seroconversion for the PT and FHA antigens positively correlated with the administered challenge dose.

### Baboon infection studies

Many animal models have been used to study *Bordetella* infections over the years, including mice, rabbits, guinea pigs, dogs, ferrets, and newborn piglets (39). Although many models replicate certain aspects of the infection, only the olive baboon (*Papio anubis*) infection model developed by the U.S. team of Dr. Tod Merkel (40–42) reproduces adequately the full spectrum of pertussis disease observed in humans. The baboon weanling infection model recapitulates the natural catarrhal pertussis transmission phase and the subsequent whooping cough observed in humans. The advantages of this model compared to human infection studies include the ability to investigate disease in newborns, collect invasive samples, use controlled transmission, study primary vaccination or infection responses, and conduct proof-of-concept testing for vaccines and therapeutics before advancing into human clinical trials. Therefore, in parallel to the controlled human infection model, a baboon infection model was implemented in Europe as part of the PERISCOPE project. Vanessa Contreras (Paris-Saclay University, Paris, France) presented data on the immune response to vaccination or infection, as well as immunity to subsequent challenge. Baboons were either vaccinated with pentavalent wP-containing or hexavalent aP-containing vaccines at 2, 4, and 6 months of age or inoculated with a low dose of the B1917 challenge strain at 2 months. At 11–14 months, all baboons were challenged with a high dose of B1917 and monitored for clinical, microbiological, and immunological outcomes. CD4^+^ T-cell responses were predominantly Th17-type following wP vaccination and Th2-type following aP vaccination, while CD8^+^ T-cell responses were absent in both groups. Both vaccines protected against *B. pertussis*-induced leukocytosis after challenge, and positron emission tomography–computed tomography scans (PET-CT) revealed acute lung inflammation only in control animals. IgG responses to *B. pertussis* antigens were observed in both vaccinated and convalescent animals and remained elevated post-challenge. Notably, wP-vaccinated animals exhibited earlier and stronger Th1 responses following challenge compared to those vaccinated with the aPV. To further explore the findings from the baboon model, Roger Le Grand (Paris-Saclay University, Paris, France) and his team conducted a study on the live attenuated vaccine BPZE1, observing that infant baboons who received intranasally 10⁹ CFU of BPZE1 7 days after birth and were challenged 16 months later with the *B. pertussis* D420 did not develop leukocytosis and exhibited low nasopharyngeal colonization. Notably, the vaccine induced a strong immune response, reflected in very high serum IgG titers (43, 44)

To conclude the session, Tod Merkel (U.S. Food and Drug Administration, Silver Spring, MD, USA) discussed efforts by his team to further advance the use of the baboon infection model. To assess whether infection from direct high-dose inoculation (10^9^ CFU) is comparable to that from natural transmission, his group compared disease progression in baboons infected via direct inoculation with those exposed through close contact with infected cage mates. They found no significant differences between the two groups. Interestingly, the pathology in the baboon model was not observed in the trachea but rather in the bronchioles, deep within the lungs, suggesting that bronchiolar damage is the primary driver of disease in baboons. Using a device placed on the scapula to measure movement in three dimensions, it was determined that infected baboons that develop pertussis disease display a significantly reduced level of movement. Dr. Merkel also reported on the use of the baboon model for the identification of novel *B. pertussis* antigens to be considered for use in new vaccine formulations. In these studies, baboons were challenged with a library of transposon mutants of the D420 strain, and outcomes of colonization and transmission were determined (45).

## EPIDEMIOLOGY AND CLINICAL ASPECTS OF PERTUSSIS

Fahima Moosa (University of the Witwatersrand, Johannesburg, South Africa) presented the findings of large prospective cohort studies conducted between 2016 and 2018, involving 1,684 participants from one rural and one urban community in South Africa, where they evaluated *B. pertussis* infection incidence by nasopharyngeal swabbing of household members twice a week and followed *B. pertussis*-specific antibody dynamics in the households. An approximately 38% rate of seroconversion was observed, and for those individuals who underwent seroconversion, the elevated antibody titers lasted 1 year post-infection (46).

The COVID-19 pandemic led to a significant decline in the number of cases of several infectious diseases (47), including pertussis. A session at the meeting was dedicated to reviewing the global epidemiology of pertussis in the post-pandemic era. Representatives from eight global regions shared data, unanimously reporting that pertussis rates were remarkably low during the pandemic. Marlena Kaczmarek, from the European Centre for Disease Prevention and Control, noted a dramatic increase in pertussis cases in mid-2023. However, the increase was not uniform across all EU/EEA countries. Peter Andersen, from Statens Serum Institute, reported that pertussis cases in Denmark rapidly increased after the pandemic, with the peak observed in November 2023 (48). Nevertheless, maternal immunization reduced the incidence of cases in young children. Katerina Fabianová, from the National Institute of Public Health, Czechia, described a significant increase in pertussis cases in the Czech Republic beginning in 2024. By the end of 2024, up to 37,510 pertussis cases were reported in a population of 10.8 million people, reaching an incidence over 347/100,000, with the highest incidence of 1,328/100,000 in the cohort of patients 15 to 19 years old (https://szu.gov.cz/temata-zdravi-a-bezpecnosti/a-z-infekce/d/davivy-kasel-pertuse/aktualni-epidemiologicka-situace-ve-vyskytu-cerneho-kasle-v-cr/) .

Helen Campbell from the UK Health Security Agency reported an increase in pertussis rates in England in the summer of 2023 among all age groups. U.S. data presented by Susan Hariri, from the Centers for Disease Control and Prevention, indicated that pertussis cases were only beginning to increase in the United States. She also reported an increase in cases of *B. parapertussis* after the pandemic. Frank Beard, from the National Centre for Immunization Research and Surveillance, Australia, reported that pertussis cases and hospitalization rates in Australia were low during the pandemic, but during the last quarter of 2023 and first 2 months of 2024, a resurgence occurred. Sibongile Walaza, from the National Institute for Communicable Diseases, presented the data from South Africa, where pertussis cases peaked in December 2022. Most pertussis cases were observed in infants aged <3 months (63.7%) (46). Finally, Efrain Montilla-Escudero, from the National Institute of Health, Colombia, described pertussis outbreaks among non-immunized Indigenous populations in Colombia, reporting that there was also an overall increase in *B. parapertussis* infections detected in 2023.

### Maternal vaccination

Maternal vaccination offers a way to protect newborns from infectious diseases prior to receipt of their own vaccinations. Research indicates that pregnant women vaccinated with the Tdap (tetanus, reduced diphtheria, and acellular pertussis) vaccine can efficiently transfer *B. pertussis*-specific antibodies to their fetus during pregnancy and to their newborn through breast milk (49, 50), with published data showing high efficacy in protection of newborns from pertussis. Nevertheless, the impact of the antibodies alone and their effect on pertussis colonization and transmission is not fully defined. The status of maternal Tdap vaccination from four countries was reported. Tami Skoff (Centers for Disease Control and Prevention, Atlanta, GA, USA) outlined that maternal Tdap vaccination has been recommended in the United States since 2012, with the coverage staying around 50%. The safety and effectiveness of Tdap during pregnancy were demonstrated in infants aged <2 months, with a sustained decrease in protection observed until 1 year of age (51). Infants of immunized mothers also have less severe pertussis if they are infected. An evaluation of immune responses to Tdap in pregnant mothers peaked with aP vaccine as infants showed that their immune responses were reduced, compared to those who had been primed with wP vaccine in infancy (52).

Helen Campbell reported that England first recommended Tdap vaccination during pregnancy in 2012 in response to a severe pertussis outbreak. Since then, the vaccine has been advised for every pregnancy between 20 and 32 weeks of gestation. Estimated maternal Tdap effectiveness against confirmed infant pertussis disease, hospitalization, and death was around 90% or higher after the program introduction (53). Maternal Tdap uptake decreased from 74.7% in December 2017 (54) to 59.5% in December 2023. Efforts are underway to increase the coverage. Nicholas Wood (The Children’s Hospital at Westmead, Westmead, NSW, Australia) outlined that maternal Tdap was funded in Australia and New Zealand since 2018. Since the COVID-19 pandemic has ended, both Australia and New Zealand have seen a significant rise in pertussis cases, including three infant pertussis deaths in New Zealand, despite levels of all-age pertussis notifications being low. Maternal Tdap vaccination rates are around 80% in Australia and 60% in New Zealand. A population-based cohort study of maternal Tdap in Australia was associated with lower risk of *B. pertussis* infection among infants through 8 months of age (55). Shelly Bolotin (Public Health Ontario, Toronto, Ontario, Canada) reported on the situation in Canada, where maternal Tdap was recommended in 2018, with provincial and territorial implementation between 2018 and 2022. Maternal vaccine coverage was 65% nationally in 2021. After low pertussis incidence during the pandemic, there has been an increase in cases in 2023 and into 2024.

### Modeling of vaccination impact

Mathematical and epidemiological models play a crucial role in understanding the impact of vaccines on disease transmission, immunity, and public health outcomes. These models help guide the policy decisions and optimize immunization programs. This session focused on exploring various models of pertussis vaccination. Meagan C. Fitzpatrick (University of Maryland School of Medicine, Baltimore, MD, USA) presented a dynamic transmission model capable of estimating the potential public health impact of next-generation pertussis vaccines (56). This model is calibrated using age-stratified US pertussis incidence and observed age-specific vaccine effectiveness. Assessing vaccine effectiveness against infection and disease for both current and next-generation vaccines is critical in establishing the value of newer vaccines. According to the model presented, the effectiveness of aPV against infection is estimated to be 20%–30%. The presented model evaluated a vaccine-induced improvement in protection against infection, duration of the protection, or both. However, it can be adapted to also estimate the impact of increased maternal vaccination coverage, improvements in booster or primary doses, and other factors.

Julie Toubiana (Pasteur Institute of Paris, Paris, France) presented an epidemiological model to assess changes in protection against pertussis disease resulting from a shift in pertussis vaccine schedule—from a 3 + 1 schedule (2, 3, and 4 months, with a booster at 16–18 months) to a 2 + 1 schedule (2 and 4 months, with a booster at 11 months)—in France in 2013. Their findings indicate that the new 2 + 1 schedule resulted in a shorter duration of protection. For instance, 3 years after vaccination, the relative risk of developing pertussis was nearly twice as high under the 2 + 1 schedule compared to the 3 + 1 schedule (57). Continuing the discussion on infant vaccination, a separate cohort study analyzed symptomatic infants under 6 months of age diagnosed with pertussis between 2008 and 2019 (58). Researchers found that PRN-producing strains were associated with an increased risk of fulminant pertussis. Since aPV contains PRN, its use appears to favor the emergence of PRN-deficient isolates, suggesting that aPV may play a role in driving *B. pertussis* evolution toward reduced virulence in infants.

Further exploring infant vaccination, Pejman Rohani (University of Georgia, Athens, GA, USA) presented an age-structured mathematical model of pertussis transmission, vaccination, and immunity. This model incorporates the dynamics of primary vaccine failure, waning of immunity, and incomplete protection. Based on data collected in Sweden from 1997 to 2021, the model estimated that DTaP (diphtheria, tetanus, and acellular pertussis) and TdaP vaccines exhibit some degree of “leakiness” but might provide some longer-lasting protection.

To model the effects of both primary and maternal vaccination, Michael Briga (Max Planck Institute for Infection Biology, Berlin, Germany) presented findings from a systematic review of the relative risk of pertussis in infants born to mothers who received maternal aP vaccination versus unvaccinated mothers, combined with a new mathematical model assessing long-term impacts (59). The study revealed that maternal immunization is highly beneficial for newborns. While modest levels of vaccine blunting in infant antibody responses to primary vaccination are possible, the benefits of maternal immunization outweigh this potential drawback. The model suggests that maternal immunization will continue to provide effective protection for unvaccinated newborns, reinforcing current public health recommendations.

## EVOLUTION, GENOMIC ASPECTS, AND REGULATION OF VIRULENCE IN *BORDETELLA*

This session provided novel insights into key areas of *Bordetella* research, including the identification of newly isolated strains from primates, vaccine-driven evolutionary changes in certain *B. pertussis* strains, and additional findings on gene expression dynamics within *Bordetella* species. Valerie Bouchez (Pasteur Institute of Paris, Paris, France) presented the most comprehensive genomic analysis of *B. parapertussis* to date, involving whole-genome sequencing analysis of 242 isolates collected between 1937 and 2019 from France, the USA, and Spain. Notably, high levels of PRN deficiency were observed, particularly among isolates obtained since 2007. In *B. pertussis*, PRN deficiency is driven by selection pressure exerted by immunity induced by aPV (60). *B. parapertussis* may also be subject to such selection pressure, although Wolfe et al. showed that O-antigen protects it from aPV-induced antibodies (61). Sarah Cameron (University of Bath, Bath, UK) described genomic analyses of *B. pertussis* that identified a 24 kb region distinguishing older pre-vaccine-era strains from contemporary strains associated with the aP vaccine era. This region contains genes involved in nicotinic acid degradation, which contribute to the faster *in vitro* growth observed in strains containing this region. Analysis to identify the link between *nic* genes, growth, and vaccination is ongoing.

There were also discussions on the characterization of novel *Bordetella* strains and the development of new models to study *Bordetella* infections. Additional insights were shared on the regulation of the BvgAS system, as well as the characterization of a newly identified virulence-regulated operon in *Bordetella* species. Tracy Nicholson (U.S. Department of Agriculture, IA, USA) genetically and phenotypically typed nineteen *B. bronchiseptica* strains obtained from baboons and rhesus monkeys, provided by Tod Merkel. Sequencing showed that two isolates from the baboons were more distantly related to all others, stretching the overall diversity of the “classical *Bordetella.*” Western blot analysis using serum generated against each isolate showed divergence that led to some loss of antibody cross-reactivity with FHA and PRN. A novel O-antigen production encoding locus, loss of the *cyaA*, *bcfA,* and the type VI secretion system (T6SS) genes were identified in the two isolates from baboons. This comparative analysis broadens our understanding of the genetic diversity in *B. bronchiseptica*. Eric Harvill (University of Georgia, Athens, GA, USA) tackled a broad theme of how Bordetellae evolved some of its remarkable abilities, such as the capacity to survive intracellularly and to ascend the Eustachian tube to colonize the middle ear to cause otitis media. Using *Bordetella pseudohinzii*, a murine acute otitis media (AOM) model was established (62). Using this model, *B. bronchiseptica (63*) and *B. pertussis* were also shown to colonize the middle ear, thus expanding our understanding of their pathogenesis. Finally, research on neonatal immunity and maternal antibody transfer to the offspring revealed that neonatal immunity can be highly effective in rapidly clearing a *B. pertussis* mutant lacking PT. When PT is produced, neutrophils are paralyzed, and bacterial growth can occur despite immune cell accumulation (64).

Deborah Hinton (National Institutes of Health, USA) described the role of a noncoding RNA, S17, in regulating the expression of BP2158, an activator of the alternative Sigma54 component of RNA polymerase (RNAP). It was revealed that regulation by S17 is highly conserved among the *Burkholderiales*, suggesting a common mechanism of regulating RNAP composition among these bacteria. This regulation plays a critical role in shaping the gene expression profile of *B. pertussis* under conditions relevant to infection (65). Branislav Vecerek (Institute of Microbiology, Prague, Czechia) explored small RNA detection. Based on the observation that the chaperone Hfq is required for virulence (66) and using RNA Interaction by ligation sequencing (RIL-seq), twenty novel small transcripts were identified in *B. pertussis*. One of them, Pred285, was conserved and dependent on Hfq. Additionally, *in vitro* assays showed that the mutant lacking Pred285 has reduced cytotoxicity against human macrophages (THP-1) and decreased biofilm formation compared to the wild-type. Loïc Coutte (Pasteur Institute of Lille, Lille, France) used complementary approaches of RNA-seq and ChIP-seq to interrogate the complex regulation of virulence-activated genes (*vags*) and virulence-repressed genes (*vrgs*) by BvgA and RisA. In these studies, BvgA required phosphorylation to bind genomic DNA and functioned to promote the expression of most *vags* either directly or indirectly. RisA displayed cooperative regulation of activity by both phosphorylation and c-di-GMP and directly regulated most *vrgs*. In addition, promoters of 164 genes were found to harbor RisA-binding sites but were not found to be regulated in RNA-seq studies, including 9 of the 15 annotated *Bordetella* sigma factors (67). Finally, Federico Sisti (University of La Plata, La Plata, Argentina) discussed the role of the second messenger cyclic di-GMP (c-di-GMP) in *B. bronchiseptica* physiology. The intracellular levels of c-di-GMP are regulated by a complex network of components. Notably, he reported that BvgR—a protein containing an EAL domain typically found in c-di-GMP-specific phosphodiesterases (PDE)—lacks the amino acid residues required for PDE activity. Nevertheless, BvgR is capable of inhibiting biofilm formation and flagellin expression, while promoting T3SS expression and cytotoxicity (68).

## *BORDETELLA* VIRULENCE FACTORS AND PATHOGENESIS

Numerous projects presented at the Symposium focused on advancing our understanding of the roles of various virulence factors and regulatory mechanisms in the development and progression of *Bordetella* infection. Rajendar Deora (The Ohio State University, OH, USA) presented new findings on *B. pertussis* polysaccharide Bps, a secreted poly-β-1,6-N-acetylglucosamine exopolysaccharide, and a key virulence factor. In addition to promoting colonization of the mouse respiratory tract and enhancing resistance to complement-mediated clearance (69), Bps also serves as a surface shield and decoy to evade human antimicrobial peptides (70). Deora also introduced a novel model for studying *Bordetella* biofilm formation, utilizing differentiated primary human bronchial ciliated epithelial cells at an air-liquid interface (71). Jana Kamanová (Institute of Microbiology, Prague, Czechia) reported on the Bsp22 protein, which forms the needle tip filament of the *Bordetella* Type 3 secretion system (T3SS). She presented super-resolution imaging data using fluorophore-labeled nanobodies, revealing that Bsp22 filaments can extend over 2 µm on abiotic surfaces without growth control, whereas during host infection, filament growth is tightly regulated. These findings highlight the dynamic adaptability of the *Bordetella* T3SS.

Two studies offered new insights into the biogenesis and function of FHA. Christopher S. Hayes (University of California, Santa Barbara, CA, USA) challenged the current model of FHA secretion, presenting intriguing data in support of the concept that the FhaB precursor protein of FHA does not solely undergo cleavage but rather delivers its C-terminal domain into host cells, in a manner similar to bacterial contact-dependent inhibition systems (CDI), which deliver their toxic C-terminal domains into competing neighboring bacterial cells. Based on structural homology with CdiA, the FhaB of adherent *B. bronchiseptica* was shown to deliver a grafted heterologous C-terminal nuclease toxin domain into eukaryotic cells. Work in progress indicates that FhaB harbors a C-terminal microtubule-binding domain, which may possibly get delivered by FhaB into the cilia of airway epithelial cells, facilitating *B. bronchiseptica* adhesion to the cilia of epithelial cells and migration of bacteria down the ciliary forest to the apical surface of the cell. Ladislav Bumba (Institute of Microbiology, Prague, Czechia) presented results obtained with a *B. pertussis-*expressing FhaBΔECT protein that lacks the C-terminal domain of FhaB (ECT) and has lost the capacity to colonize the nasal cavity of mice and bind the ciliated primary human nasal epithelial cells differentiated *ex vivo*. Adhesion of *B. pertussis* to human nasal ciliated cells could also be inhibited by anti-ECT serum, underpinning the importance of the ECT domain in the adhesin function of FhaB/FHA.

Hongbin Li (University of British Columbia, Vancouver, Canada) provided key insights into the folding mechanism of CyaA toxicity. Specifically, he showed that the Repeats-in-Toxin (RTX) domain of CyaA follows a strictly ordered template folding process dictated by calcium binding, which progresses from the C- to N-terminus. Disrupting this sequence prevents proper toxin function, thus opening potential therapeutic avenues for targeting CyaA.

Martin Zmuda (Institute of Microbiology, Prague, Czechia) described data on how the BteA Type III-secreted effector of *B. bronchiseptica* induces host cell necrosis by disrupting calcium homeostasis. Consequently, BteA causes rapid permeabilization of cellular membranes and fragmentation of the mitochondria and endoplasmic reticulum, leading to cell death. A genome-wide CRISPR-Cas9 screen failed to identify host suppressor factors of BteA-mediated cytotoxicity, suggesting that BteA acts pleiotropically or that it targets potentially redundant host pathways (72).

Expanding on *Bordetella* toxin diversity, Seema Mattoo (Purdue University, West Lafayette, IN, USA) introduced the highly conserved Fic protein of *Bordetella* (termed BbFic) as a potential novel virulence factor. She showed that BbFic is endowed with guanylyltransferase activity, distinct from the ATP-dependent activity of most Fic proteins. Additionally, BbFic exhibits a protein toxin activity by post-translational GMPylation of putative target proteins involved in maintaining DNA supercoiling and integrity. Michael Gollan (Northumbria University, Newcastle, UK) reported the identification of two orphan proteins, BP1251 and BP1252, that are homologous to B subunits of AB_5_ toxins and were found to contribute to *B. pertussis* adhesion and host colonization. David Rickert (University of Maryland, Baltimore, MD, USA) reported that up or downregulation of production of tracheal cytotoxin (TCT), a *B. pertussis-*shed muramyl peptide fragment of bacterial peptidoglycan, can modulate the pathology in the lungs and proposed that *B. pertussis* manages the host response by regulating TCT release. Dacine Osmani (Pasteur Institute of Lille, Lille, France) demonstrated that nasal-mimicking conditions (35°C) trigger a transcriptomic response in *B. pertussis*, characterized by increased expression of PT and FHA compared to standard 37°C conditions.

Jason McLellan (The University of Texas at Austin, TX, USA) outlined the approach to structure-based design of vaccines against the SARS-CoV-1 and SARS-CoV-2 viruses (73) and recent efforts on gaining structure-based understanding of pertussis vaccine antigen immunogenicity, aiming at boosting their potency, while optimizing the size and form (74, 75).

Françoise Jacob-Dubuisson (Pasteur Institute of Lille, Lille, France) presented studies on a newly identified virulence-regulated operon in *B. pertussis*, which is a member of the ribosomally synthesized, post-translationally modified peptide (RiPP) family of metallophores. The *B. pertussis* RiPP, termed “bufferin,” was expressed and modified by genes in the *bp2924-2927* operon. This operon was positively regulated by BvgAS and produced a copper-binding RiPP. Data generated using a RiPP knockout strain under copper restricted conditions suggested a role for *B. pertussis* RiPP in copper storage or the shuttling of copper between import and utilization by heme-copper oxidases. This work highlighted the necessity of copper utilization by *B. pertussis (*76).

María Eugenia Rodríguez (University of La Plata, La Plata, Argentina) discussed key differences in the intracellular environments of *B. pertussis* within human macrophages and respiratory epithelial cells. It appears that bacterial intracellular trafficking, nutrient availability, and stress conditions differ significantly between these two cell types. The intracellular survival of *Bordetella* bacteria within macrophages, but not within epithelial cells, critically depends on the modulation of the bactericidal activity of the cells by PT and AC toxins (77–79). These findings suggest that respiratory epithelial cells provide a more favorable environment for long-term bacterial persistence, whereas macrophages, though less suited for persistence, may serve as vehicles to transport live bacteria to other niches.

## BIOLOGY AND IMMUNOLOGY EXPLORATION LEADING TO A LIVE ATTENUATED NASAL PERTUSSIS VACCINE

In his keynote lecture, Camille Locht (Pasteur Institute of Lille, Lille, France) summarized his 40 years of seminal research on *B. pertussis* biology and immunology, initiated by cloning, sequencing, and deciphering of the structure of the operon encoding PT production (80, 81). The elucidation of the enzymatic mode of action of PT eventually led to development of genetically inactivated pertussis toxoids for use in pertussis vaccines (82). Dr. Locht also outlined his studies on the major adhesin FhaB/FHA, the structure-function relationships underlying BvgAS-mediated regulation of *B. pertussis* virulence, and immune responses to *B. pertussis* infections in both mice and humans. His lecture culminated in the description of the design, preclinical testing, and progress of clinical studies of the live attenuated *B. pertussis* BPZE1 strain. This intranasal vaccine against *B. pertussis* is currently heading into phase III clinical trials and has the potential to significantly restrict *B. pertussis* morbidity and transmission (83).

## IMMUNITY AND VACCINATION

Immunization with aPV effectively prevents severe pertussis in infants but does not confer long-lasting protection from *B. pertussis* infection and colonization of the airways, allowing increased pathogen circulation within the highly aP-vaccinated population. To address this limitation, scientists are actively exploring strategies to improve pertussis vaccines. Diane Gbesemete and Stephanie Noviello (University of Southampton, Southampton, UK, and ILiAD Biotechnologies, Weston, FL, USA) presented the phase 2b results of the intranasal live attenuated pertussis vaccine BPZE1 trial. Intranasal vaccine administration prevented nasal colonization in up to 98.7% of *B. pertussis*-challenged participants, which is critical to halt transmission. Furthermore, the BPZE1 vaccine had an excellent safety profile; only a few cases of mild adverse events, such as fever, headache, fatigue, or runny nose, were reported, which were not different in the frequency and severity to the placebo control group (83). In a parallel effort, Daniela Hozbor (University of La Plata, La Plata, Argentina) reviewed the progress toward a pertussis outer membrane vesicle (OMV)-based vaccine (84). The OMV vaccine, which had a better safety profile than a wPV in mice, induced Th1- and Th2-type cellular immune responses and IgG1 and Ig2a antibody responses in mice and protected mouse lungs from *B. pertussis* infection. The pertussis OMVs also acted as an adjuvant, boosting immune responses to an aPV (85).

Exploring new technologies to develop vaccines, Heath Damron (West Virginia University, Morgantown, WV, USA) described the use of an mRNA platform for a new vaccine against pertussis (86). Immunization of mice with a diphtheria, tetanus, and pertussis (multiple antigens) mRNA vaccine protected against *B. pertussis* infection of the lungs and trachea, but not the nose. The mRNA vaccine also prevented pertussis disease in a coughing rat model (87). Addition of mRNA coding for the chemokine CXCL13 served as a genetic adjuvant to boost antibody responses to the pertussis mRNA vaccine.

Also exploiting the use of different adjuvants to enhance vaccine immunogenicity, Purnima Dubey (The Ohio State University, OH, USA) presented work on optimizing adjuvant-antigen administration strategies to enhance mucosal immunity against *Bordetella* species. Her findings showed that the Th1/Th17 polarizing adjuvant BcfA (88) with the current aPV significantly improved Th1/Th17 responses to *Bp* antigens. Notably, her studies highlighted that a combined delivery strategy, using an intramuscular prime followed by an intranasal boost, conferred heightened protection against *B. pertussis* lung and nasal colonization in a murine model (89). Additionally, employing unbiased immuno-peptidomics approaches, her team identified novel highly conserved *B. pertussis* antigens presented on MHC Class II glycoproteins that were recognized by CD4^+^ tissue resident memory T (T_RM_) cells in the nose and lungs of mice.

Finally, studies have revealed the potential benefits of combining pertussis vaccines with other vaccines to enhance efficacy. Dimitri Diavatopoulos (Radboud University Medical Centre, Nijmegen, The Netherlands) defined a role for the inactivated polio virus vaccine (IPV) containing viral RNA, which acted as an adjuvant to enhance innate immune responses in individuals immunized with DTaP-IPV (90). A comparison of gene expressions in blood before and one day after immunization revealed significant upregulation of anti-viral, especially type-1 interferon, gene expression signatures. These innate immune responses were mediated by TLR-activation of p38 in myeloid dendritic cells and correlated with persistence of *B. pertussis* specific serum IgG. Buddy Creech (Vanderbilt University, Nashville, TN, USA) presented transcriptomics data that identified differences in Toll-like receptor signaling pathways and inflammation in infants immunized with wPV and aPV. Sonia McAlister (University of Western Australia, Nedlands, Australia) reported that repeated maternal Tdap boosters of wPV-primed and aPV-primed mothers enhanced immune interference to the childhood vaccine in 6-month-old infants. **J**olanda Brummelman (RIVM, Bilthoven, The Netherlands) showed that functional T-cell responses to an aPV booster vaccine retained the phenotype of the priming immunization and were stronger and markedly TH2 skewed in children 7–14 years old.

The innate and adaptive immune responses to *Bordetella* species in animal models and human studies were also discussed. Karen Scanlon (University of Maryland School of Medicine, Baltimore, MD, USA) tackled cooperative defense strategies and adverse host responses to gain an understanding on how the early-life immune system responds to *B. pertussis*. Her work focused on how age influences disease outcomes, particularly based on the observation that neonates can survive despite carrying extremely high bacterial loads. Her findings revealed that neonates have higher tryptophan availability, which supports immunosuppression and creates a favorable environment for *Bordetella* species to achieve such high colonization levels. Disruption of host tryptophan metabolism leads to immune activation and increased bacterial clearance, which can provide a novel therapeutic target especially in young infants highly susceptible to severe pertussis manifestations. Ciaran Skerry (University of Maryland School of Medicine, Baltimore, MD, USA) using infant and adult mice showed that pertussis induces long-term pathophysiology and described the role of the inflammation-amplifying receptor TREM-1 in exacerbating collagen deposition and atelectasis in this process (91). Colleen Sedney (University of Georgia, Athens, GA, USA) continued the discussion on neonatal immunity. Her findings suggested that PT employs a two-target mechanism to decrease neutrophil responses in neonate mice: by targeting the CXCL1/CXCR2 signaling pathway and by targeting complement activation to suppress neutrophil recruitment (92). Regarding mouse models, Monica Cartelle Gestal (Louisiana State University, Shreveport, LA, USA) described a role for the BtrS sigma factor in attenuating immune responses to three *Bordetella* species and that infection with *B. bronchiseptica* having the *btrS* gene deleted results in recruitment of eosinophils into airway mucosa, which supported bacterial clearance and adaptive immunity (93). To conclude the section of Immunity and Vaccination, Alessandro Sette (La Jolla Institute for Immunology, La Jolla, CA, USA) discussed the importance of identifying immunodominant CD4^+^ T cell antigens and showed that a high-throughput bioinformatics screen of the *B. pertussis* genome, along with functional assays, identified new epitopes recognized by T cells from individuals immunized with wPV (94).

Caitlín Ní Chasaide (Trinity College Dublin, Dublin, Ireland) reported that immunization of mice with aPV vaccines induces the expansion of *B. pertussis* antigen-specific IL-10-secreting regulatory T cells. These cells suppress the induction of respiratory CD4^+^ T_RM_ cells that are essential for mucosal protection against nasal infections with *B. pertussis* in mice. She showed that blocking IL-10 signaling during aP immunization enhanced bacterial clearance in the nose and enhanced Th17-type tissue T_RM_ cells. Furthermore, the addition of stimulator of interferon genes (STING) or Toll-like receptor 2 (TLR2) agonists as adjuvants enhanced the efficacy of the aPV against nasal infection in mice. Overall, this study indicates that the use of alum as an adjuvant in aP vaccines impairs protective immune responses by increasing IL-10 regulatory T cell signaling.

In his thought-provoking keynote lecture, Kingston Mills (Trinity College Dublin, Dublin, Ireland) presented data from the mouse neonatal infection model, which provided an explanation for the origin of neurological complications often observed in infants with severe pertussis infection. *B. pertussis* infections in neonatal mice resulted in blood-brain barrier disruption, dissemination of *B. pertussis* bacteria into the brain, and concomitant immune cell infiltration and microglia activation, resulting in defective synaptic pruning and subsequent autism spectrum disorder symptoms in convalescent mice (95). These findings further highlight the importance of deciphering the immune mechanisms, enabling clearance of *B. pertussis* infection from the nasopharyngeal mucosa, and identifying effective approaches for eliciting protective mucosal immune responses with the next-generation pertussis vaccines. The Mills team has previously discovered in the mouse infection model that *Bordetella* antigen-specific nasal CD4^+^ T_RM_ cells that secrete IL-17 play a key role in directing protective mucosal immune responses, enabling Siglec F^+^ neutrophil infiltration and clearance of *B. pertussis* infection from the nasal mucosa (96). Recent studies confirmed that such CD4^+^ T_RM_ cells are expanded in the nasopharyngeal mucosa of wP-vaccinated humans, whereas aP-vaccinated humans have significantly lower numbers of these cells in the upper airway mucosa (97). It remains to be validated in humans whether aPV triggers IL-10-secreting regulatory T cells that suppress protective CD4^+^ T_RM_ cells specific for *B. pertussis* antigens. The acquired knowledge on the mechanism of protective immunity has already been utilized by the Mills lab to develop a promising prototype of a novel mucosal pertussis vaccine. This vaccine contains antibiotic-inactivated whole *B. pertussis* bacterial cells (AIBP). When administered intranasally in booster experiments, the AIBP vaccine was able to overcome the blunting effect of the aP vaccine on CD4^+^ T_RM_ cell expansion and conferred sterilizing immunity in the lower and upper airways of mice, highlighting its potential for clinical testing in humans.

Toward the end of the meeting, Xin-Xing Gu (National Institute of Allergy and Infectious Diseases of the NIH, Bethesda, MD, USA) presented the pertussis research resources (https://www.niaid.nih.gov/research/resources?f%5B0%5D=field_division%3A12&f%5B1%5D=field_research_stage%3A264) and support to extramural investigators and vaccine developers (both U.S. and non-U.S. investigators, who are welcome to apply for grants and contracts to conduct basic and translational research, preclinical product development, and clinical evaluation through NIH- and NIAID-Wide Funding Opportunity Announcements and Requests for Proposals (http://grants.nih.gov/grants/guide/parent_announcements.htm).

The final session was a panel discussion involving representatives of pertussis vaccine developers and manufacturers, including epidemiologists, clinicians, and pertussis researchers. Discussions focused on the options, strategies, pitfalls, and future directions for developing novel ideal pertussis vaccines. These vaccines should aim to save lives and protect infants and older children from severe pertussis and provide robust protection of the nasopharyngeal mucosa against *B. pertussis* infection across the age spectrum. Such vaccines are urgently needed to effectively control the transmission of Bordetellae and ultimately reduce pertussis-related infant mortality and morbidity in older individuals.

## CONCLUSION

The 14th *Bordetella* Symposium offered invaluable insights into the recent advancements of our understanding of *Bordetella* species and their biology and impact on health. Key highlights included groundbreaking findings from the PERISCOPE Consortium, discussions on the human and baboon infection models, updates on vaccine development efforts against whooping cough, and analyses of epidemiological trends and disease prevalence across various regions. Further topics explored the effectiveness of maternal vaccination strategies and advances in understanding the pathogenesis and biology of the pathogen and groundbreaking new insights into the immunology of *B. pertussis* infections in animal models. Despite this significant progress, much remains to be uncovered about *Bordetella* biology and infection mechanisms. To continue building on these advancements, the scientific community looks forward to the 15th International *Bordetella* Symposium, scheduled to take place in Nashville, TN, USA, in 2026. This next meeting will undoubtedly serve as another crucial milestone in the collective effort to combat *Bordetella*-related diseases.
